# Arl4c promotes the growth and drug resistance of pancreatic cancer by regulating tumor-stromal interactions

**DOI:** 10.1016/j.isci.2021.103400

**Published:** 2021-11-03

**Authors:** Xin Chen, Yanzhen Zhang, Weikun Qian, Liang Han, Wei Li, Wanxing Duan, Zheng Wu, Zheng Wang, Qingyong Ma

**Affiliations:** 1 Department of Otorhinolaryngology-Head and Neck Surgery, The First Affiliated Hospital of Xi'an Jiaotong University, Xi'an 710061, China; 2 Department of Hepatobiliary Surgery, The First Affiliated Hospital of Xi'an Jiaotong University, Xi'an 710061, China

**Keywords:** Biological sciences, Cell biology, Functional aspects of cell biology, Cancer

## Abstract

Emerging evidence suggests that ADP-ribosylation factor like-4c (Arl4c) may be a potential choice for cancer treatment. However, its role in pancreatic cancer, especially in tumor-stroma interactions and drug resistance, is still unknown. In the current study, we examined the proliferation and drug resistance effect of Arl4c on pancreatic cancer cells. Furthermore, we explored the contribution of Arl4c high expression in pancreatic stellate cell (PSC) activation. We found that high Arl4c expression is associated with cell proliferation, drug resistance, and PSC activation. In detail, Arl4c regulates connective tissue growth factor (CTGF) paracrine, further induces autophagic flux in PSCs, resulting in PSC activation. TGFβ1 secreted by activated PSCs enhances cancer cell stem cell properties via smad2 signaling, further increasing cell drug resistance. YAP is an important mediator of the Arl4c-CTGF loop. Taken together, these results suggest that Arl4c is essential for pancreatic cancer progression and may be an effective therapeutic choice.

## Highlights

• High Arl4c expression is correlated with PSCs activation and drug resistance
• Yap-CTGF-mediated autophagy is required for Arl4c-related PSCs activation
• Paracrine TGFβ1 of PSCs plays pivotal role in drug resistance of pancreatic cancer cells

## Graphical abstract

**Figure:**
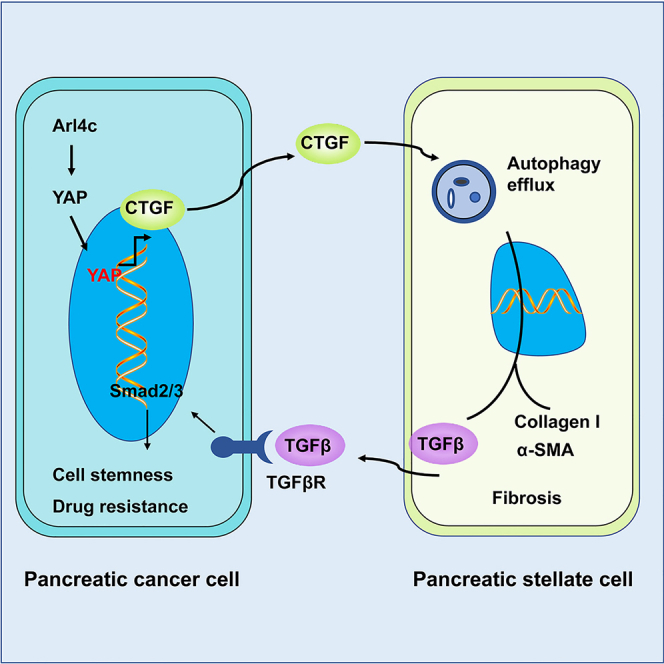

## Introduction

Pancreatic ductal adenocarcinoma (PDAC) is one of the most lethal cancers in the world, with a 5-year survival rate of less than 9% (Siegel et al., 2020). PDAC is set to be the most common cause of cancer-related death within the next decade (Rahib et al., 2014). With the improvement of medical instruments, an increasing number of patients are diagnosed with pancreatic cancer at an early tumor stage. However, at present, only approximately 10% of patients are diagnosed with standard surgically resectable pancreatic cancer (Strobel et al., 2019). Local invasion and drug resistance remain two obstacles resulting in a poor cancer prognosis. Pancreatic cancer is characterized by a severe desmoplastic reaction that consists of activated pancreatic stellate cells (PSCs), lymphocytes, macrophages, vascular endothelial cells, extracellular matrix (ECM) and other components, creating a complex tumor microenvironment that promotes tumor growth, metastasis, and drug resistance (Dougan, 2017). Recently, the interaction between the tumor and tumor microenvironment has attracted much research interest (Thomas and Radhakrishnan, 2019). However, the underlying mechanism is still poorly understood.

PSCs are the most prominent cell type responsible for the development of the extensive fibrotic stroma in pancreatic cancer, resulting in a highly hypoxic and nutrient-poor tumor environment. Under normal conditions, pancreatic stellate cells are in a quiescent status characterized by a star shape and vitamin A enrichment and are located at the basolateral aspect of acinar cells or surrounding the perivascular and periductal areas in the healthy pancreas (Meng et al., 2019). With pancreatic tumorigenesis, quiescent pancreatic stellate cells are activated by various cytokines secreted by cancer cells or other stromal cells, such as sonic hedgehog (shh), connective tissue growth factor (CTGF), platelet-derived growth factor (PDGF), vascular endothelial growth factor (VEGF), and transforming growth factor β (TGFβ1), and transform into myofibroblast-like cells with increased abilities to synthesize and secrete matrix and certain cell growth factors (Dunér et al., 2011; Karger et al., 2008). Activated PSCs are present in PanIN specimens in the early stage of pancreatic carcinogenesis, suggesting that PSCs may play an important role in the development of pancreatic cancer (Arumugam et al., 2017). To date, the interactions between PSCs and cancer cells, including immunomodulation and metabolic reprogramming, have been verified to create a favorable microenvironment for the carcinogenesis, invasion, metastasis, and drug resistance of pancreatic cancer (Dalin et al., 2019; Parker et al., 2020; Sousa et al., 2016). However, the detailed mechanism is not well understood.

ADP-ribosylation factor (Arf) like-4c (Arl4c) is a small GTP-binding protein belonging to the ARL4 family of proteins, which includes Arl4a, Arl4c, and Arl4d (Chen et al., 2019). During organic development, Arl4c is essential for the growth factor-dependent formation of epithelial tubular structures (Matsumoto et al., 2014). Arl4c is expressed at high levels in tumors and exerts its oncogenic role in the tumorigenesis, growth, and metastasis of diverse cancers, such as colorectal cancer, lung cancer, and hepatocellular carcinoma (Chen et al., 2019; Fujii et al., 2015; Harada et al., 2019). The underlying mechanism involves the activation of Rac1, inhibition of Rho, and upregulation of PIK3CD (Chen et al., 2019; Harada et al., 2019). However, Arl4c was recognized as a tumor suppressor in ovarian cancer, as higher Arl4c expression inhibits cell migration and indicates a favorable prognosis (Su et al., 2015). Recently, using immunohistochemistry, Arl4c was identified as a biomarker for a worse prognosis and a novel therapeutic target in endometriosis-associated ovarian cancer (Wakinoue et al., 2019). Based on these studies, the function of Arl4c shows tumor specificity and pathology specificity. All these studies focus on cancer cells, and the role of Arl14c in tumor stromal cells and tumor-stromal interactions remains unknown.

In this report, we aimed to explore the role of Arl4c in pancreatic cancer growth and drug resistance, as well as PSC activation, further determine its effect on tumor-stromal interactions, and reveal the underlying mechanism.

## Results

### Arl4c is required for the proliferation and drug resistance of pancreatic cancer

**Figure 1:**
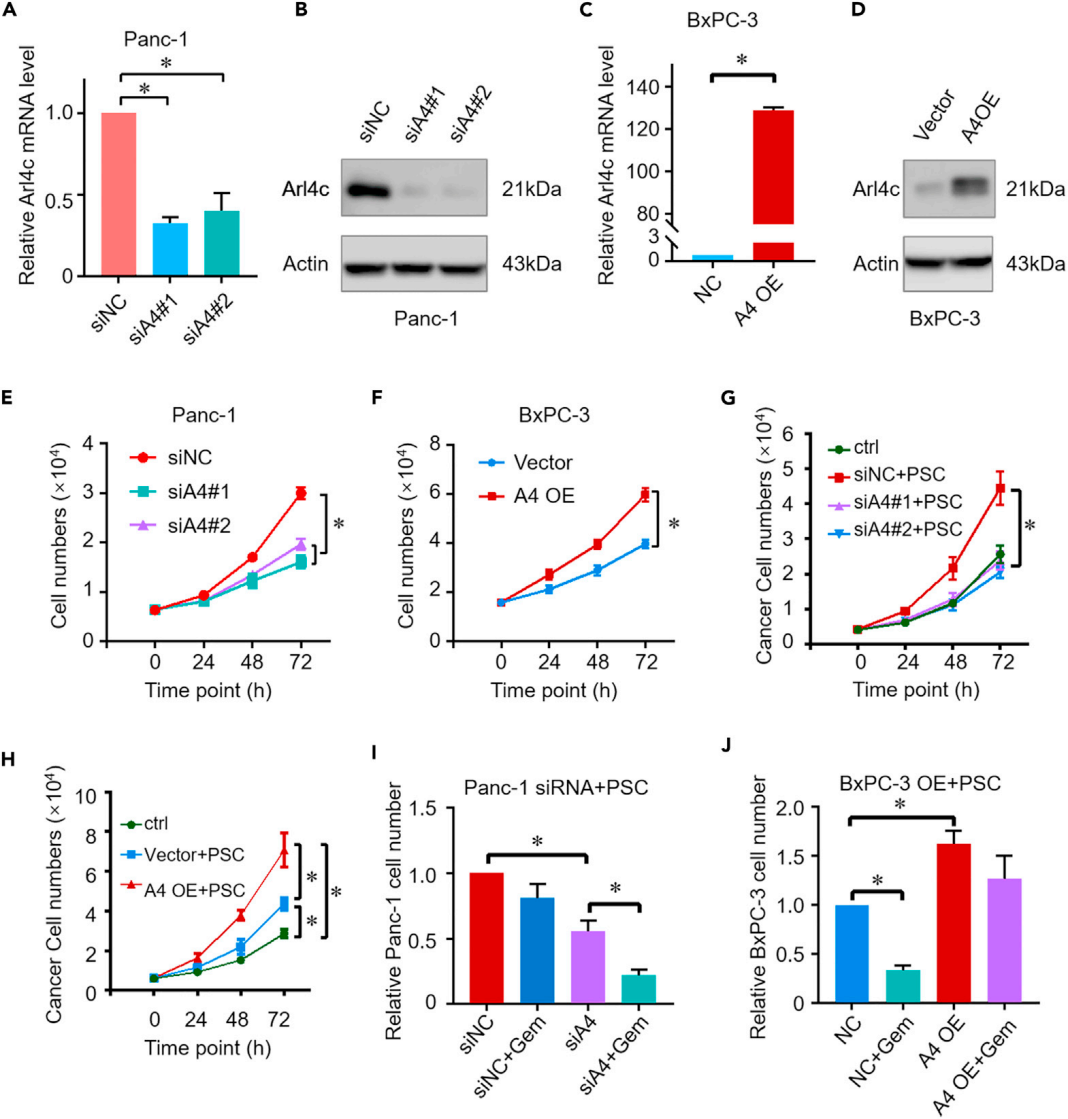
Arl4c promotes cell growth and drug resistance in pancreatic cancer (A) Quantification of Arl4c mRNA expression in Panc-1 cells with or without Arl4c knockdown and analysis via Student's t test. ∗, p < 0.05. Data are represented as mean ± SEM (B) Levels of the Arl4c protein in Panc-1 cells with or without Arl4c knockdown, as analyzed using western blotting. (C) Quantification of Arl4c mRNA expression levels in BxPC-3 cells with or without Arl4c overexpression and analysis via Student's t test. ∗, p < 0.05. Data are represented as mean ± SEM. (D) Expression of the Arl4c protein in BxPC-3 cells with or without Arl4c overexpression, as analyzed using western blotting. (E and F) Proliferation of Panc-1 or BxPC-3 cells with different Arl4c expressions levels in a 3D culture system tested using an MTT assay analyzed via Student's t test. Data are represented as mean ± SEM. ∗, p < 0.05. (G and H) Proliferation of Panc-1 or BxPC-3 cells with different Arl4c expression levels when cocultured with PSCs tested using an MTT assay. Data are represented as mean ± SEM. ∗, p < 0.05 by one-way ANOVA test. (I and J) Numbers of Panc-1 or BxPC-3 cells with the indicated treatments when cocultured with PSCs tested using the MTT assay. Gemcitabine, 4.5 μM, for Panc-1 treatment and 3 μM gemcitabine for BxPC-3 cells treatment. Data are represented as mean ± SEM. ∗, p < 0.05 by one-way ANOVA test.

Arl4c expression was silenced by two individual siRNAs to explore the role of Arl4c in cell growth. These two siRNAs decreased Arl4c expression at both the mRNA and protein levels in Panc-1 cells (Figures 1A and 1B). Conversely, Arl4c expression was successfully increased by the Arl4c overexpression plasmid in BxPC-3 cells (Figures 1C and 1D). Cells were then seeded in 6-well plates, and cell numbers were calculated at different time points. Neither downregulation nor upregulation of Arl4c expression affected cell proliferation (data not shown). Tumors are complex entities composed of cancer cells, stromal cells, and other components. We then seeded these cells in 3D Matrigel and carried out a cell proliferation test. To our surprise, Arl4c knockdown retarded Panc-1 cell proliferation (Figure 1E). In contrast, Arl4c overexpression increased the number of BxPC-3 cells compared with the control group (Figure 1F). These results imply that Arl4c may play a role in the tumor microenvironment. Therefore, we cultured cancer cells with PSCs, an important component of PDAC, using an indirect coculture system. Arl4c knockdown inhibited the proliferation of Panc-1 cells even on a two-dimensional (2D) plastic dish (Figure 1G). Conversely, overexpression of Arl4c promoted the proliferation of BxPC-3 cells when cultured with PSCs (Figure 1H). We further explored whether Arl4c affected the drug resistance of pancreatic cancer cells. In Panc-1 cells, downregulation of Arl4c did not affect gemcitabine resistance without PSC coculture (data not shown). However, the cell number decreased robustly in the coculture system (Figure 1I). In contrast, Arl4c upregulation promoted gemcitabine resistance in BxPC-3 cells in the coculture system (Figure 1J). Based on these results, Arl4c promotes cell proliferation and drug resistance in pancreatic cancer.

### Overexpression of Arl4c in pancreatic cancer cells promotes PSC activation

**Figure 2:**
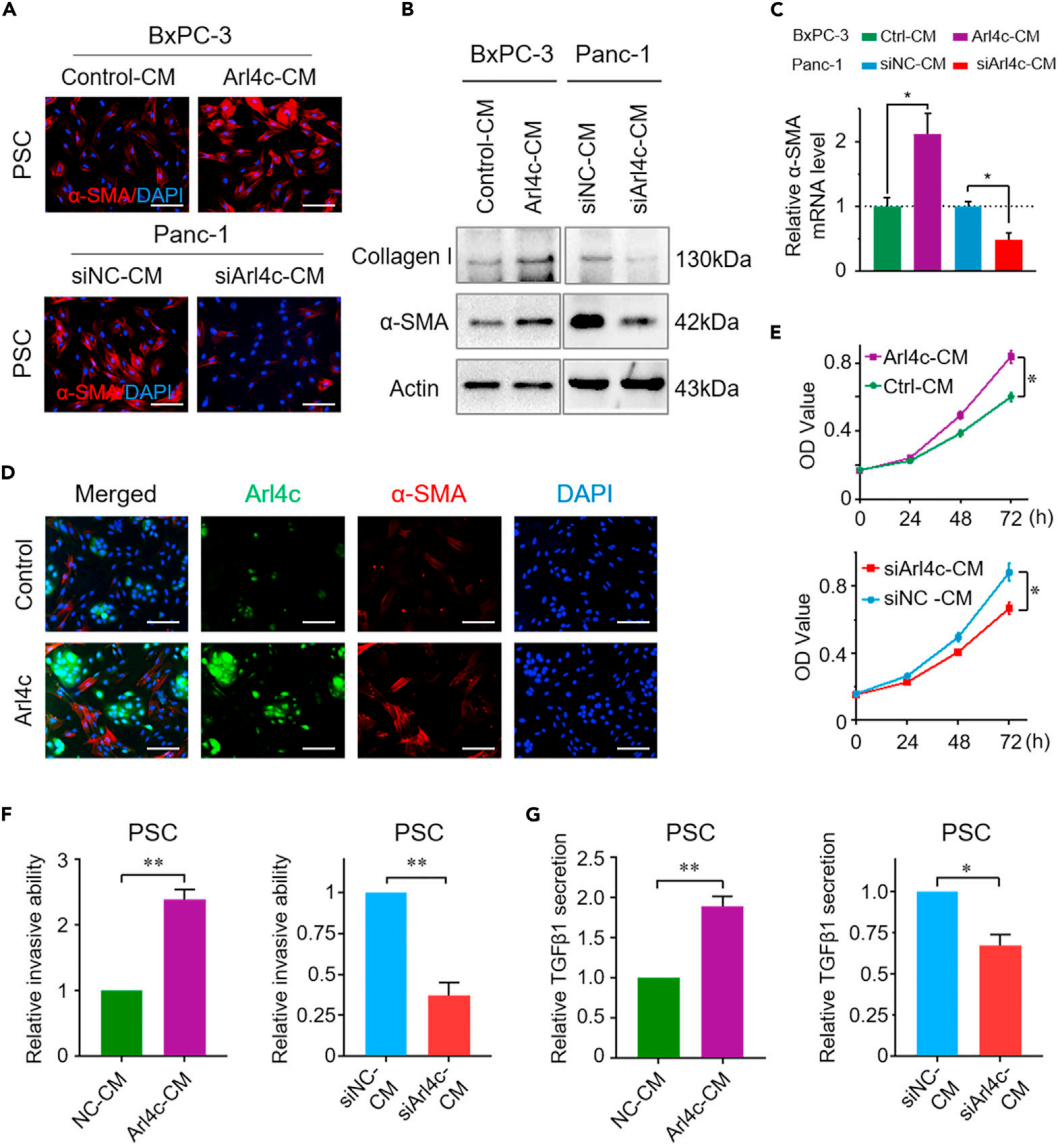
Inhibition of Arl4c impairs PSC activation (A) Representative images of immunofluorescence staining of PSCs stained with α-SMA when cultured with conditioned medium from cancer cells with Arl4c overexpression (upper panel) or knockdown (lower panel) (bar, 100 μm). (B) Western blotting assay of collagen I and α-SMA levels in PSCs treated as described in Figure 2A. (C) Relative α-SMA mRNA expression level in PSCs treated as described in Figure 2A. Data are represented as mean ± SEM. ∗, p < 0.05 by one-way ANOVA test. (D) BxPC-3 cells with or without Arl4c overexpression were cultured with PSCs for 48 h, and immunofluorescence staining was performed to detect α-SMA and Arl4c expression in these cells (scale bar, 100 μm). (E) Pancreatic cancer cells with Arl4c overexpression (upper panel) or knockdown (lower panel) were cultured for 36 h, and then cell-conditioned medium was collected, centrifuged, and used to treat PSCs. The cell number was detected at the indicated time points using the MTT assay. Data are represented as mean ± SEM. ∗, p < 0.05 by Student's t test. (F) PSCs were treated with cancer cell (with Arl4c overexpression or knockdown)-conditioned medium for 24 h, and cell invasion was measured using the Transwell invasion assay. Relative cell invasion is shown. Data are represented as mean ± SEM. ∗, p < 0.05 and ∗∗, p < 0.01 by Student's t test. (G) PSCs were treated as described in Figure 2F for 36 h, and then the cells were cultured in normal medium for another 36 h. TGFβ1 concentrations in the supernatant were determined using an ELISA. Data are represented as mean ± SEM. ∗, p < 0.05 and ∗∗, p < 0.01 by Student's t test.

The diverse effects of Arl4c on cell proliferation in 2D and 3D culture systems prompted us to hypothesize that Arl4c may play a role through PSCs. Thus, we treated PSCs with cancer cell-conditioned medium (CM). Compared with the control group, PSCs treated with Arl4c-overexpressing cancer cell CM (Arl4c-CM) showed a higher expression level of αSMA, as identified using immunofluorescence (IF) staining (Figure 2A). Meanwhile, the expression of αSMA and collagen I was also elevated at both the protein and mRNA levels when PSCs were cultured in Arl4c-CM (Figures 2B and 2C). In contrast, Arl4c knockdown conditioned medium (siArl4c-CM) decreased αSMA and collagen I expression at both protein and mRNA levels compared with the control group (Figures 2A–2C). Cancer cells and PSCs were cultured in the same plate, and IF was performed to more directly show the effect of Arl4c on PSC activation. Arl4c was mainly expressed in the cytoplasm of tumor cells, and no Arl4c staining was found in PSCs. The PSC activation level was elevated upon culture with Arl4c-overexpressing BxPC-3 cells, as revealed by αSMA staining (Figure 2D). Moreover, Arl4c-CM promoted PSC proliferation and invasion (Figures 2E and 2F), accompanied by a higher level of TGFβ1 secretion by PSCs (Figure 2G). In contrast, siArl4c-CM inhibited PSC proliferation and invasion compared with the control group (Figures 2E and 2F). Meanwhile, PSCs treated with siArl4c-CM showed less TGFβ1 secretion (Figure 2G). These results indicate that Arl4c expression in cancer cells promotes PSC activation.

### CTGF mediates the pro-desmoplastic reaction of Arl4c

**Figure 3:**
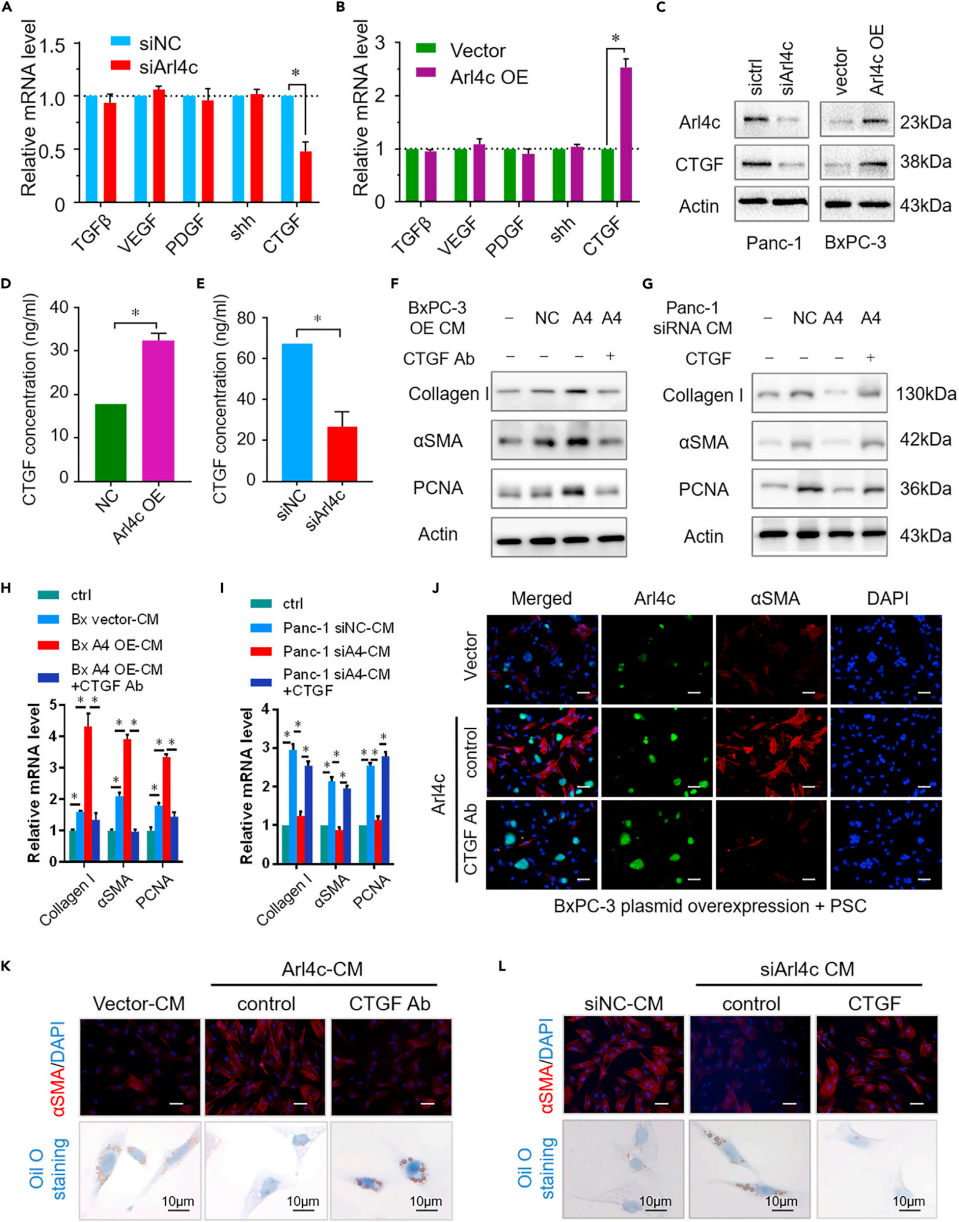
CTGF inhibition reverses Arl4c-mediated PSC activation (A) Relative levels of the TGFβ1, VEGF, PDGF, shh, and CTGF mRNAs in Panc-1 cells with or without Arl4c knockdown. Data are represented as mean ± SEM. ∗, p < 0.05 by Student's t test. (B) Relative levels of the TGFβ1, VEGF, PDGF, shh, and CTGF mRNAs in BxPC-3 cells with or without Arl4c overexpression. Data are represented as mean ± SEM. ∗, p < 0.05 by Student's t test. (C) Total protein was extracted from pancreatic cancer cells with Arl4c knockdown or overexpression, and immunoblotting was performed. (D) Supernatants of BxPC-3 cells with or without Arl4c overexpression were collected, and the CTGF concentration was determined using an ELISA. Data are represented as mean ± SEM. ∗, p < 0.05 by Student's t test. (E) Supernatants of Panc-1 cells with or without Arl4c knockdown were collected, and the CTGF concentration was determined using an ELISA. Data are represented as mean ± SEM. ∗, p < 0.05 by Student's t test. (F and G) PSCs were treated with cancer cell-conditioned medium and immunoblotting was performed. (H and I) Relative levels of the collagen I, α-SMA, and PCNA mRNAs in PSCs treated as indicated. Data are represented as mean ± SEM. ∗, p < 0.05 by one-way ANOVA test. (J) BxPC-3 cells exposed to the indicated treatments were cultured with PSCs for 48 h, and immunofluorescence staining was performed to detect α-SMA and Arl4c expression in these cells (scale bar, 50 μm). (K) PSCs were treated with the indicated cell-conditioned medium in the presence or absence of the CTGF-neutralizing antibody. Immunofluorescence (scale bar, 50 μm) and oil O staining (scale bar, 10 μm) were performed, and representative images are shown. (L) PSCs were treated with the indicated cell-conditioned medium in the presence or absence of CTGF, immunofluorescence (scale bar, 50 μm) and oil O staining (scale bar, 10 μm) were performed, and representative images are shown.

According to previous studies, cancer cells secrete diverse factors, such as sonic hedgehog, TGFβ1, VEGF, CTGF, and PDGF, which promote PSC activation (Li et al., 2014; Liu et al., 2012). Here, we tested whether the Arl4c intervention affected the mRNA levels of these factors. In Panc-1 cells, Arl4c knockdown significantly decreased CTGF mRNA levels, with no obvious effects on sonic hedgehog, TGFβ1, VEGF, or PDGF, as evidenced by the qRT-PCR results (Figure 3A). In contrast, Arl4c overexpression increased CTGF mRNA expression, with little effect on sonic hedgehog, TGFβ1, VEGF, and PDGF expression (Figure 3B). Meanwhile, a similar change was observed in CTGF protein levels in cancer cells (Figure 3C). More importantly, a higher concentration of CTGF was detected in the supernatants of Arl4c-overexpressing BxPC-3 cells than in control BxPC-3 cells, as determined using an ELISA (Figure 3D). Conversely, Arl4c knockdown resulted in a lower CTGF level in the supernatants of Panc-1 cells (Figure 3E). Next, we explored whether CTGF is the main factor responsible for Arl4c-mediated PSC activation. Therefore, an additional CTGF Ab (10 μg/ml) was added to Arl4c-overexpressing BxPC-3 supernatants and then used to treat PSCs. As shown in Figures 3F, 3H, and 3K, BxPC-3-Arl4c-CM increased collagen I, αSMA, and PCNA expression, changes were abolished by the CTGF Ab treatment at both the mRNA and protein levels. Moreover, the additional CTGF (10 ng/ml) intervention reversed the inhibitory effect of siArl4c-CM on PSC activation (Figures 3G, 3I, and 3L). Moreover, BxPC-3 cells with different Arl4c expression levels and PSCs were seeded in a direct coculture system, and the CTGF Ab intervention was applied. High Arl4c expression induced PSC activation, whereas the CTGF Ab treatment successfully inhibited PSC activation caused by Arl4c, as revealed by αSMA staining, with no effect on Arl4c expression (Figure 3J). Quiescent PSCs are characterized by large amounts of stored lipids that could be used for the tumor energy supply upon activation (Saison-Ridinger et al., 2017). Here, compared with the control group, Arl4c-CM treatment eliminated PSC lipid storage, whereas the addition of the CTGF Ab rescued lipid storage in PSCs (Figure 3K). Conversely, siArl4c-CM increased PSC lipid storage, which was abolished by the CTGF intervention (Figure 3L). Taken together, these data suggest that Arl4c regulates PSC activation via CTGF in a paracrine manner.

### YAP inhibition reverses Arl4c-mediated CTGF paracrine signaling

**Figure 4:**
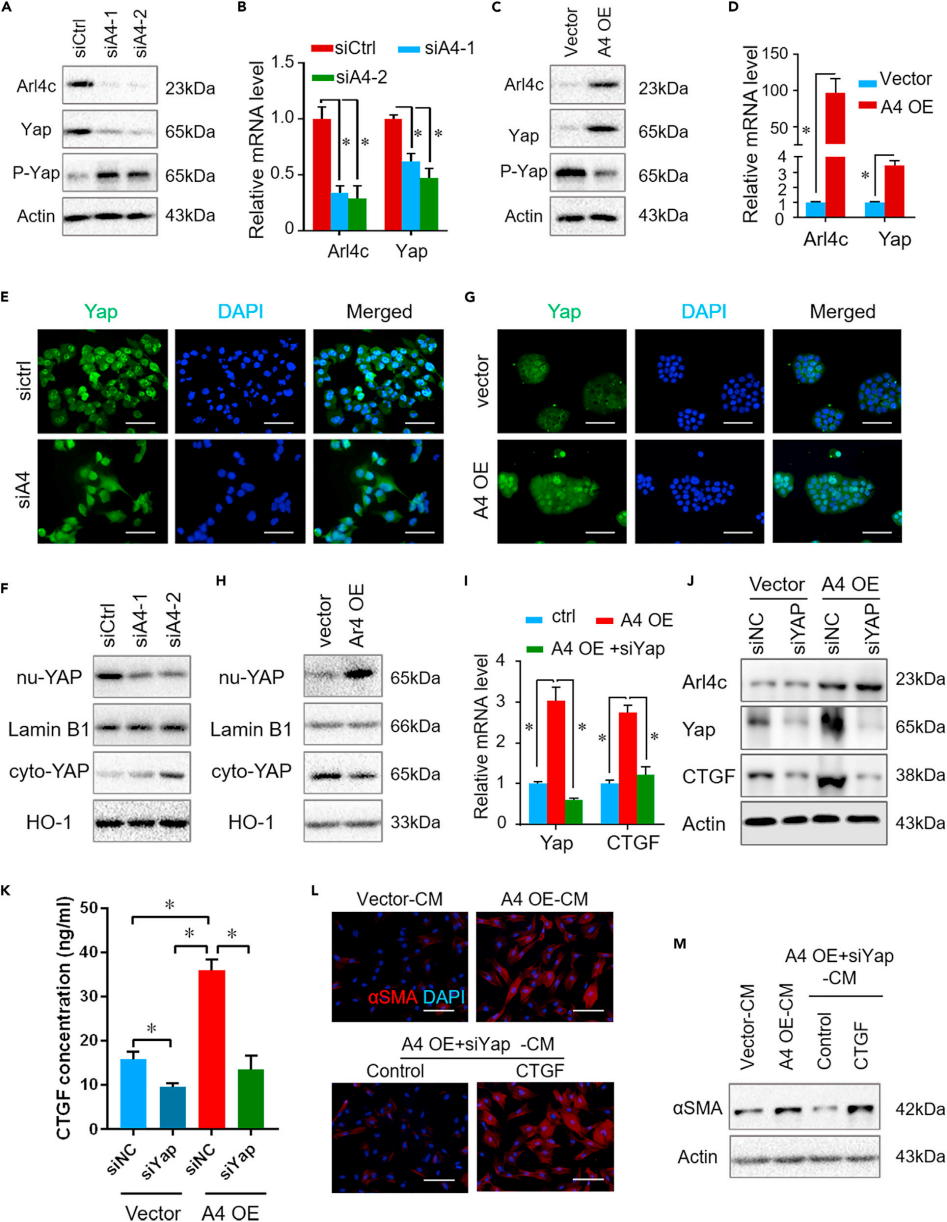
YAP1 is an important mediator of the Arl4c-CTGF loop (A) Arl4c was downregulated in Panc-1 cells, and immunoblotting was performed. (B) Levels of the YAP1 and Arl4c mRNAs in Panc-1 cells with Arl4c knock down. Data are represented as mean ± SEM. ∗, p < 0.05 by one-way ANOVA test. (C) Arl4c was overexpressed in BxPC-3 cells, and immunoblotting was performed. (D) Levels of the YAP1 and Arl4c mRNAs in BxPC-3 cells overexpressing Arl4c. Data are represented as mean ± SEM. ∗, p < 0.05 by one-way ANOVA test. (E) Representative image of immunofluorescence staining for Yap1 in Panc-1 cells with or without Arl4c knockdown (scale bar, 100 μm). (F) Arl4c was knocked down, cytoplasmic and nuclear proteins were extracted, and immunoblotting was performed. (G) Representative image of immunofluorescence staining for Yap1 in BxPC-3 cells with or without Arl4c overexpression (scale bar, 100 μm). (H) Arl4c was overexpressed, cytoplasmic and nuclear proteins were extracted, and immunoblotting was performed. (I) Levels of the YAP1 and CTGF mRNAs in cells with Arl4c overexpression or knock down were measured using qRT-PCR. Data are represented as mean ± SEM. ∗, p < 0.05 by one-way ANOVA test. (J) BxPC-3 cells with different expression levels of Arl4c were transfected with or without the Yap1 siRNA for 48 h, and immunoblotting was performed. (K) BxPC-3 cells with different expression levels of Arl4c were transfected with or without the Yap1 siRNA for 48 h, and the CTGF concentration in the supernatant was determined using ELISA. Data are represented as mean ± SEM. ∗, p < 0.05 by one-way ANOVA test. (L and M) Conditioned medium from BxPC-3 cells with the indicated treatments was collected and centrifuged and added to PSCs for 48 h with or without the additional CTGF intervention. Immunofluorescence staining and immunoblotting were performed (scale bar, 100 μm). Data are represented as mean ± SEM. ∗, p < 0.05.

Our previous research verified that YAP promotes pancreatic tumor progression by regulating tumor-stroma interactions (Jiang et al., 2018). Here, we explored whether YAP was an important mediator of the Arl4c-CTGF loop. Knockdown of Arl4c in Panc-1 cells decreased YAP expression at both the protein and mRNA levels, as revealed by western blotting and qPCR (Figures 4A and 4B). In contrast, Arl4c overexpression in BxPC-3 cells robustly enhanced total YAP expression at both the protein and mRNA levels (Figures 4C and 4D). In addition, knockdown of Arl4c resulted in increased p-YAP levels, and overexpression of Arl4c reduced p-YAP levels (Figures 4A and 4C). More importantly, knockdown of Arl4c resulted in cytoplasmic localization of YAP, whereas Arl4c overexpression led to nuclear localization of YAP, as evidenced by IF staining and immunoblotting (Figures 4E–4H). Based on these data, Arl4c regulated YAP expression at both the mRNA and protein levels. We used an siRNA targeting YAP in Arl4c-overexpressing BxPC-3 cells and performed experiments to further confirm that YAP is required for Arl4c-mediated CTGF regulation. Arl4c overexpression increased YAP and CTGF expression at both the mRNA and protein levels, changes were reversed by YAP knockdown in BxPC-3 cells (Figures 4I and 4J). Moreover, knockdown of YAP reduced the levels of CTGF secreted from Arl4c-overexpressing BxPC-3 cells (Figure 4K). Furthermore, we used the supernatants of BxPC-3 cells to treat PSCs. As shown in Figures 4L and 4M, Arl4c-overexpressing BxPC-3-CM promoted PSC activation, whereas the supernatant of BxPC-3 cells with Arl4c overexpression plus YAP knockdown inhibited PSC activation. More importantly, additional CTGF intervention rescued PSC activation, as revealed by western blotting and IF staining (Figures 4L and 4M). Together, these data suggest that Arl4c regulates PSC activation via YAP-mediated CTGF paracrine signaling pathway.

### Autophagy efflux induced by CTGF paracrine signaling regulates PSC activation

**Figure 5:**
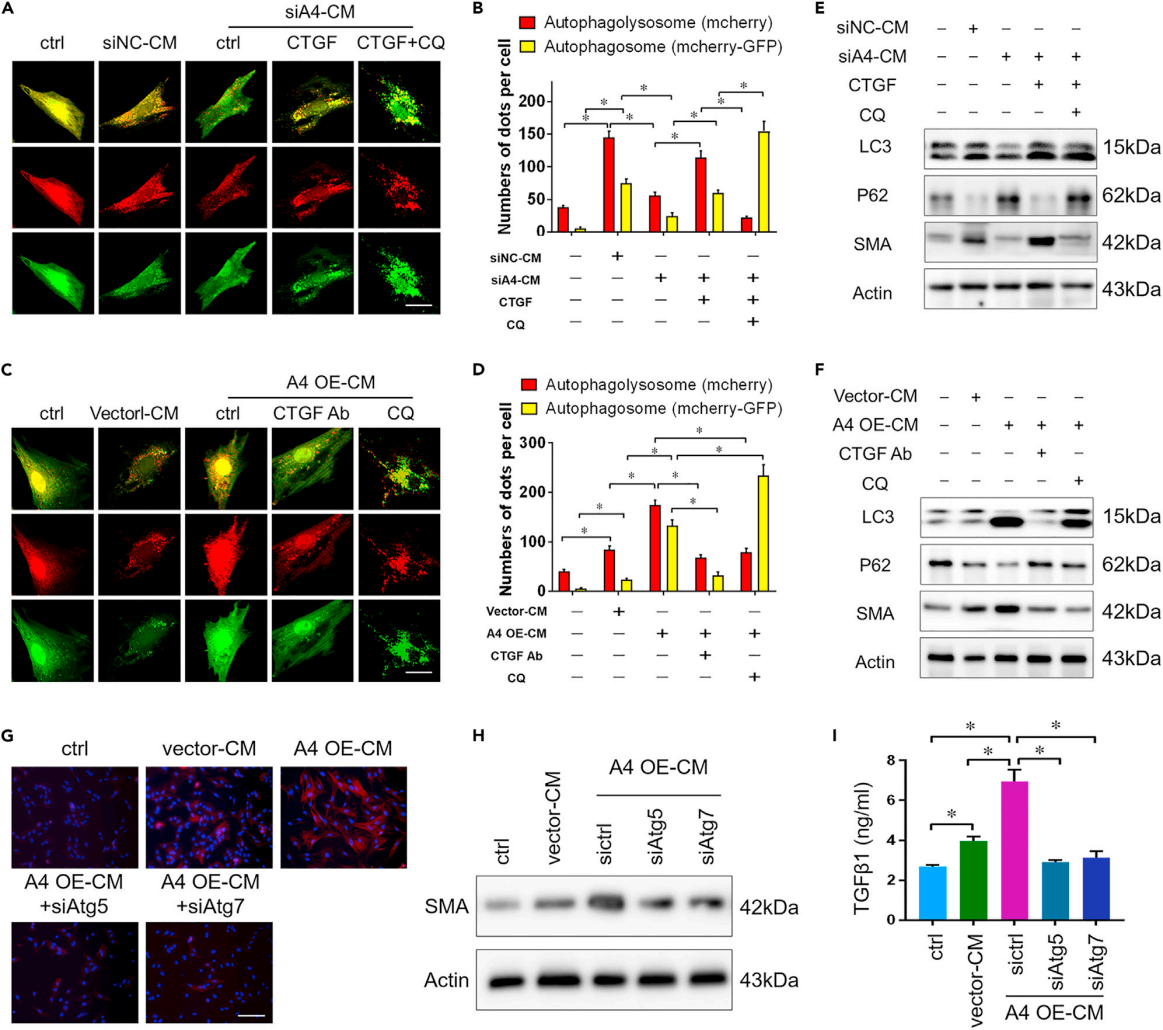
Autophagy inhibition reverses CTGF-induced PSC activation (A) Conditioned medium from Panc-1 cells with or without Arl4c knockdown was collected, centrifuged, and stored until use. PSCs were transfected with the mCherry-GFP-LC3 adenovirus, and 48 h later, cells were treated with cancer cell-conditioned medium in the presence or absence of the additional CTGF or CQ treatment for another 48 h. Cells were then fixed, and images were captured using a confocal microscope (scale bar, 10 μm). (B) Quantification of red and yellow fluorescent dots representing mCherry-EGFP-LC3 in Figure 5A. Data are represented as mean ± SEM. ∗, p < 0.05 by one-way ANOVA test. (C) Conditioned medium from BxPC-3 cells with or without Arl4c overexpression was collected, centrifuged, and stored until use. PSCs were transfected with mCherry-GFP-LC3 adenovirus, and 48 h later, cells were treated with cancer cell-conditioned medium in the presence or absence of the CTGF-neutralizing antibody or additional CQ treatment for another 48 h. Cells were then fixed, and images were captured using a confocal microscope (scale bar, 10 μm). (D) Quantification of red and yellow fluorescent dots representing mCherry-EGFP-LC3 in Figure 5C. Data are represented as mean ± SEM. ∗, p < 0.05 by one-way ANOVA test. (E) PSCs without mCherry-GFP-LC3 transfection were treated as described in Figure 5A, and immunoblotting was performed. (F) PSCs without mCherry-GFP-LC3 transfection were treated as described in Figure 5C, and immunoblotting was performed. (G and H) PSC cells were transfected with the ATG5 siRNA or ATG7 siRNA for 24 h and then treated with conditioned medium from BxPC-3 cells with different expression levels of Arl4c. After 48 h, immunofluorescence staining (G) and immunoblotting (H) were performed (scale bar, 100 μm). (I) PSCs were treated as described in Figure 5G, and levels of secreted TGFβ1 in supernatants were detected using an ELISA. Data are represented as mean ± SEM. ∗, p < 0.05 by one-way ANOVA test.

We next elucidated the underlying mechanism between CTGF and PSC activation. Autophagy has been reported to be associated with tumor fibrosis (Cui et al., 2020; Endo et al., 2017). An mCherry-GFP-LC3 adenovirus was employed to assess the effect of CTGF on PSC autophagy. Owing to the sensitivity of GFP to the acidic environment of autolysosomes, red dots represent autolysosomes, and yellow dots represent autophagosomes (You et al., 2019). PSCs transfected with this plasmid were incubated with cancer cell-derived CM, and cells were observed under a fluorescence microscope (confocal laser scanning microscope) after 48 h. Compared with the control siRNA, siArl4c-CM decreased the numbers of both autophagosomes and autophagolysosomes in PSCs, which was rescued by addition CTGF treatment (Figures 5A and 5B). In contrast, Arl4c-overexpressing BxPC-3-CM promoted PSC activation accompanied by increased numbers of red and yellow dots clustered in the cytoplasm. This promoting effect was inhibited by CTGF Ab intervention, with fewer spots of autolysosomes, as revealed by confocal laser scanning microscopy (Figures 5C and 5D). Consistent with these findings, additional CTGF treatment promoted LC3 ⅡB and αSMA expression and inhibited the expression of P62, a negative marker of cellular autophagy, in PSCs treated with siArl4c-CM (Figure 5E). Chloroquine (CQ), an inhibitor of autophagy that blocks the binding of autophagosomes to lysosomes, successfully inhibited the stimulatory effect of CTGF (Figure 5E). Compared with cells treated with Arl4c OE-CM, additional treatment with CTGF Ab inhibited cellular expression of LC3 ⅡB and αSMA (Figure 5F). Based on these data, Arl4c-mediated paracrine CTGF signaling may regulate PSC activation through its role in autophagosome biogenesis. We further explored whether knockdown of two important mediators of classic autophagy, Atg5 and aAtg7, would affect PSC activation. Knockdown of one of these two genes in PSCs reversed the effect of Arl4c OE-CM on promoting PSC activation, as revealed by IF staining and immunoblotting (Figures 5G and 5H). Meanwhile, the level of TGFβ1 secreted from PSCs was also reduced by Atg5 or Atg7 knockdown (Figure 5I). Thus, Arl4c-mediated CTGF paracrine signaling activated PSCs by inducing autophagy.

### Activated PSCs promote drug resistance in pancreatic cancer via TGFβ1/smad2 signaling

**Figure 6:**
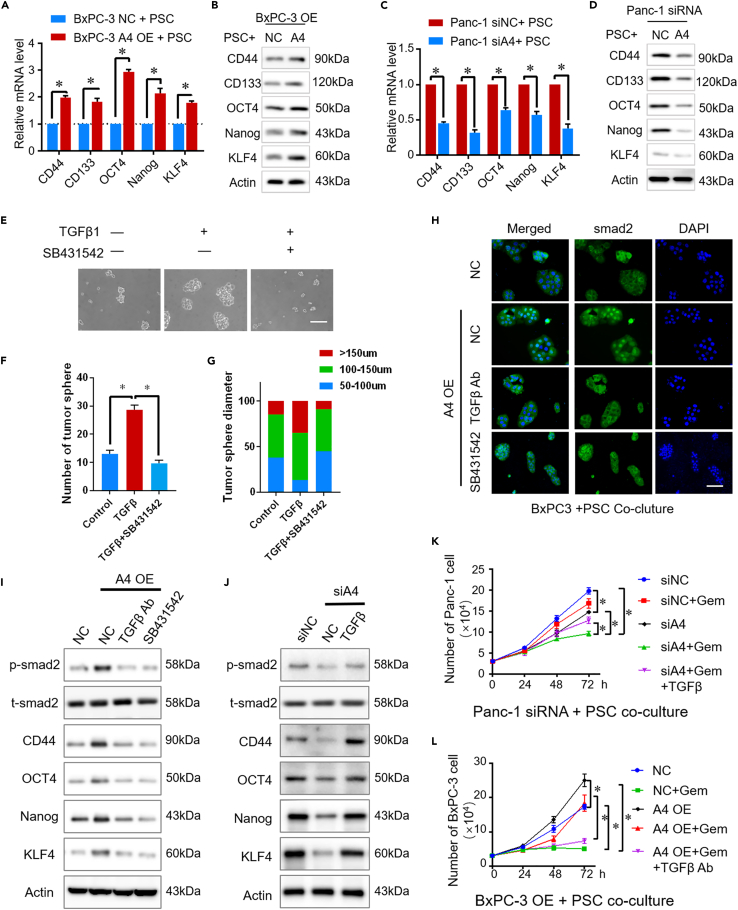
TGFβ1 promotes drug resistance by regulating cell stemness properties (A) BxPC-3 cells with different Arl4c expression levels were indirectly cocultured with PSCs for 48 h, and the mRNA levels of certain genes were determined using qRT-PCR. (B) BxPC-3 cells with different Arl4c expression levels were indirectly cocultured with PSCs for 48 h, and immunoblotting was performed. (C) Panc-1 cells with different Arl4c expression levels were indirectly cocultured with PSCs for 48 h, and the mRNA levels of certain genes were determined using qRT-PCR. (D) Panc-1 cells with different Arl4c expression levels were indirectly cocultured with PSCs for 48 h, and immunoblotting was performed. (E) BxPC-3 cells were treated with TGFβ1 (10 ng/mL) or TGFβ1 plus SB431542 (10 μM), and representative images of the tumor sphere formation assay were captured (scale bar, 100 μm). (F and G) Cells were treated as described in Figure 6E, and the number of tumor spheres was counted and plotted. The percentage of tumor spheres with diameters of 50–100, 100–150, or >150 μm was calculated and plotted. Data are represented as mean ± SEM. ∗, p < 0.05 by one-way ANOVA test. (H) BxPC-3 cells with different Arl4c expression levels were indirectly cocultured with PSCs and then treated with the indicated reagents. Immunofluorescence staining was performed on cancer cells (scale bar, 100 μm). (I and J) Cancer cells with different Arl4c expression levels were indirectly cocultured with PSCs and then treated with the indicated reagents. Forty-eight hours later, the total protein was extracted from cancer cells and immunoblotting was performed. (K and L) Cancer cells with different Arl4c expression levels were indirectly cocultured with PSCs and then treated with the indicated reagents. The numbers of cancer cells were counted at the indicated time points. Data are represented as mean ± SEM. ∗, p < 0.05 by one-way ANOVA test.

Cell stemness is reported to be associated with drug resistance in bladder cancer (Zhang et al., 2017). We detected the expression of several markers of cell stemness, such as CD44, CD133, OCT4, Nanog, and KLF4, to test whether the effect of Arl4c on promoting drug resistance in pancreatic cancer is attributed to its role in regulating cell stemness. In pancreatic cancer cells, we did not observe any obvious changes in either mRNA or protein levels of these markers when Arl4c was knocked down or upregulated compared with the control group (data not shown). Next, we used an indirect coculture system in which PSCs were seeded in the upper 0.5-μm chamber and tumor cells were seeded in the lower chamber. Cellular protein was extracted from cancer cells 48 h later. As shown in Figures 6A and 6B, in this indirect coculture system, Arl4c OE-BxPC-3 cells displayed higher expression of these stemness markers at both the mRNA and protein levels than the control group. In contrast, siArl4c-Panc-1 cells showed lower expression of these markers when cocultured with PSCs than sictrl-Panc-1 cells (Figures 6C and 6D). These data indicate that Arl4c-mediated regulation of cell stemness may be responsible for its effect on PSCs.

As shown above, PSCs cultured in Arl4c OE-CM secreted TGFβ1 at higher levels. TGFβ1, 10 ng/ml, treatment increased not only the number but also the diameter of the BxPC-3 tumor spheres, and this effect could be reversed by the TGFβ1 receptor inhibitor SB431542 (10 μM) (Figures 6E–6G). Therefore, TGFβ1 regulates pancreatic cancer cell stemness. Furthermore, we detected whether TGFβ1 secreted by PSCs activated TGFβ1/smad2 signaling in a paracrine manner in cancer cells. In this indirect coculture system, Arl4c OE-BxPC-3 cells exhibited nuclear localization of smad2, whereas control BxPC-3 cells showed a predominantly cytoplasmic localization (Figure 6H). Both the TGFβ1 neutralizing antibody (1μg/ml) and SB431542 abolished smad2 nuclear localization, implying that PSC-derived TGFβ1 activated smad2 signaling in tumor cells (Figure 6H). Compared with the control group, Arl4c OE-BxPC-3 showed increased psmad2 levels accompanied by CD44, OCT4, Nanog, and KLF4 upregulation, changes that were inhibited by the TGFβ1 neutralizing antibody (1 μg/ml) and SB431542 (Figure 6I). In contrast, in the indirect coculture system of Panc-1 cells and PSCs, Arl4c knockdown in cancer cells reduced the levels of psmad2 and cancer stemness markers, and these changes were rescued by an additional TGFβ1 treatment (Figure 6J). Based on these data, PSC-derived TGFβ1 regulates tumor stemness via TGFβ1/smad2 signaling.

We further detected the drug resistance of cancer cells in this indirect coculture system. Compared with the control group, Panc-1 cells with Arl4c knockdown exhibited decreased cell numbers upon gemcitabine treatment. However, additional TGFβ1 treatment increased cell numbers compared with the gemcitabine-treated Arl4c knockdown group, although the number was still less than the group treated with gemcitabine alone (Figure 6K). Next, BxPC-3 cells with or without Arl4c overexpression were treated with gemcitabine. Compared with the control group, Arl4c overexpression increased cell numbers upon gemcitabine treatment, whereas additional TGFβ1Ab treatment abolished gemcitabine resistance (Figure 6L). Therefore, the paracrine effects of TGFβ1 secreted after Arl4c-induced activation of PSCs promotes tumor drug resistance and stemness via TGFβ1/smad2 signaling.

### Arl4c expression correlates with αSMA expression in pancreatic cancer

**Figure 7:**
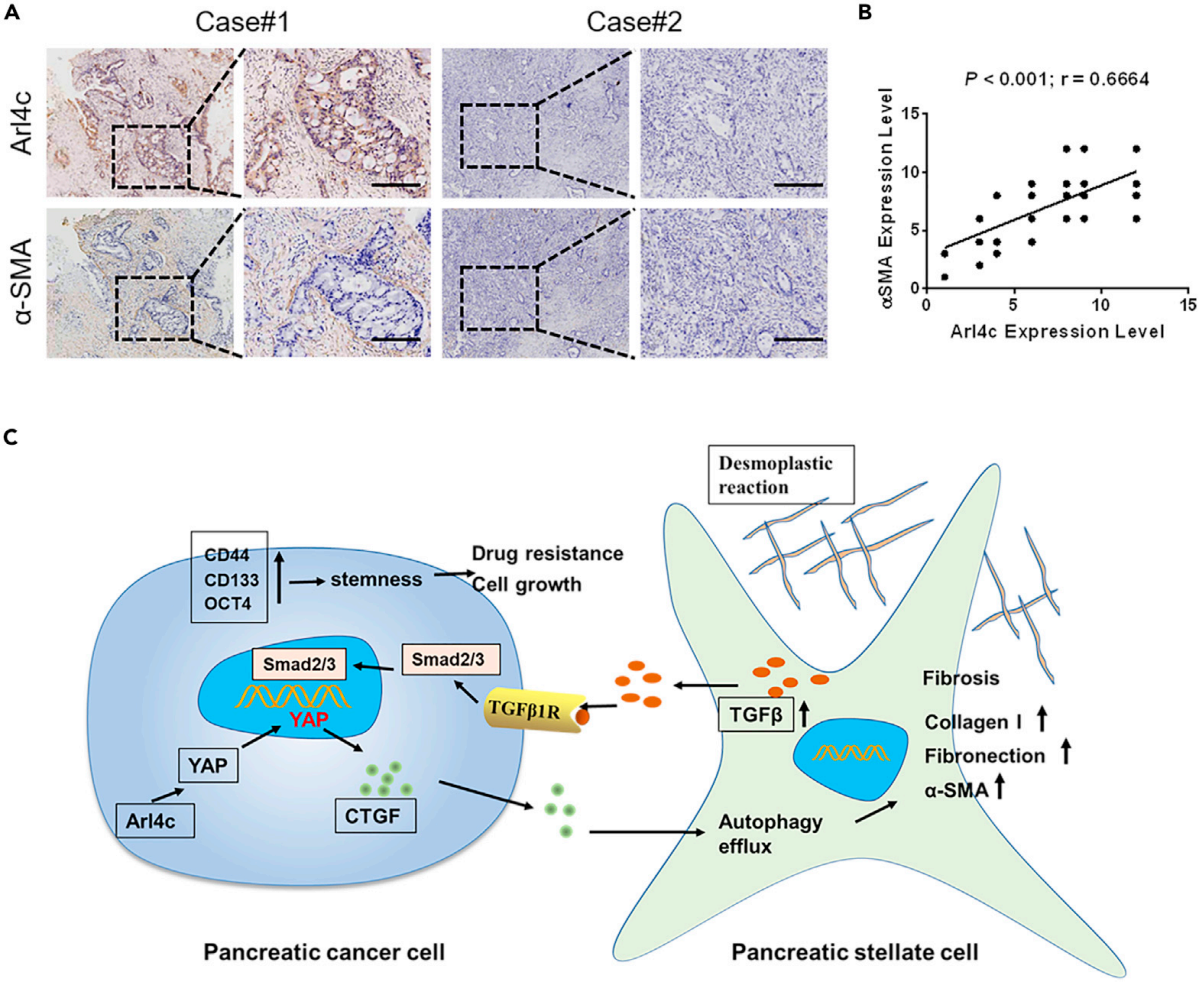
Correlation of Arl4c and αSMA expression in human PDAC specimens (A) Representative images of pancreatic cancer patients' samples showing Arl4c and αSMA expression using immunohistochemistry (scale bar, 500 μm). (B) Assessment of positive correlation between Arl4c and αSMA expression in PDAC specimens using Pearson correlation coefficient analysis. Note that the scores of some samples overlap. (C) A schematic model illustrating the molecular mechanism by which Arl4c regulates the tumor-stromal interaction.

To assess the potential clinical relevance of our findings, we detected the expressions of Arl4c and αSMA in 52 pancreatic cancer specimens. The statistics for Arl4c expression levels in different pancreatic tissue groups are shown in Table S1. PSCs in pancreatic cancer specimens with high Arl4c expression have a higher αSMA expression, whereas these in pancreatic cancer specimens with low Arl4c expression often lose αSMA expression (Figure 7A). Statistical analysis revealed that expression levels of Arl4c were significantly correlated with αSMA expression levels (r = 0.6664, P < 0.001) (Figure 7B), suggesting Arl4c correlates with tumor fibrosis in pancreatic cancer.

## Discussion

Pancreatic cancer is characterized by a severe desmoplastic reaction that is responsible for drug resistance in cancer cells, but the underlying mechanism is still largely unknown. In this study, we identified a critical role for Arl4c in pancreatic fibrosis and drug resistance via Yap 1-dependent CTGF paracrine signaling and subsequent increases in the autophagy level and activation of PSCs, which led to pancreatic cancer cell self-renewal and stemness. Our clinical and mechanistic findings establish that the Arl4c-Yap-CTGF axis is critical for pancreatic cancer progression and that treatments targeting this axis might be a potential therapeutic strategy for pancreatic cancer.

In our study, using a coculture system and cell supernatant, we found that Arl4c expression in cancer cells correlated with PSC activation. This correlation was confirmed in samples from patients with pancreatic cancer. As tumors can secrete various cell factors targeting PSCs, we selected several common factors, performed ELISAs, and identified CTGF as a potential target. YAP signaling endows cancer cells with several important roles, such as metabolic reprogramming, drug resistance, cell growth, and metastasis (Coggins et al., 2019; Er et al., 2018; Koo and Guan, 2018). Currently, its role in tumor-stromal interactions, especially in tumor fibrosis, has attracted increasing attention. Recently, researchers found that YAP inhibition in PSCs resulted in PSC deactivation (Xiao et al., 2019). Moreover, our previous study also revealed correlations between high expression of YAP in pancreatic cancer cells and pancreatic fibrosis and PSC activation, whereas the underlying mechanism is only partially understood (Jiang et al., 2018). Autophagy is reported to be associated with cardiac fibrosis and pulmonary fibrosis (Liu et al., 2017; Shirakabe et al., 2016). The effect of CTGF signaling on autophagy was reported only in several studies, and the potential mechanism may involve HIF1, Erk, and Akt regulation (Ni et al., 2020; Capparelli et al., 2012). In this report, we found Arl4c-mediated paracrine CTGF signaling promotes PSC activation, accompanied by a higher level of autophagy flux, changes that were reversed by autophagy inhibitors. YAP silencing abolished the effect of Arl4c on promoting PSC activation, which was rescued by additional CTGF treatment. As an upstream regulator of YAP, Arl4c promotes YAP expression at both the mRNA and protein levels. Of interest, although these regulatory relationships remained in the 2D culture system, we did not observe any significant effect of Arl4c on cancer cell growth, implying the existence of other regulatory mechanisms downstream of Arl4c, which require further exploration.

Desmoplastic reactions in the tumor microenvironment are considered factors supporting pancreatic cancer progression. However, as the main resource of the tumor ECM, deletion of PSCs in transgenic pancreatic cancer mice resulted in diminished survival and enhanced tumor aggressiveness (Özdemir et al., 2014). This interesting finding suggests that the role of PSCs in pancreatic cancer may depend on the tumor stage. Pancreatic cancer cells can recruit PSCs to establish an environment that promotes cancer progression (Vonlaufen et al., 2008). Moreover, local and distant cancer migration are verified to be accompanied by PSC migration (Xu et al., 2010). In the present study, high Arl4c expression not only promoted PSC proliferation but also enhanced the migratory ability of PSCs. PSC-derived CXCL12, deoxycytidine, and fibronectin were reported to promote drug resistance in pancreatic cancer; however, the underlying mechanism remains unclear (Amrutkar et al., 2019; Dalin et al., 2019; Singh et al., 2010). TGFβ1 is reported to be a versatile cytokine involved in cancer cell metastasis, drug resistance, and tumor fibrosis (Aoyagi et al., 2004; Lambies et al., 2019). In our study, Arl4c-mediated PSC activation resulted in a higher level of TGFβ1 secretion. TGFβ1 paracrine signaling by PSCs then promotes the stemness and drug resistance of cancer cells by activating smad2 signaling. The TGFβ1 neutralizing antibody abolished the effect of Arl4c on promoting the stemness and drug resistance of pancreatic cancer cells. We did not observe any significant changes in the levels of secreted TGFβ1 when Arl4c was knocked down in the cancer cells themselves, further confirming that this effect of Arl4c on promoting tumor stemness and drug resistance depends on its role in PSCs.

Arl4c is reported to be involved mainly in tumor invasion and metastasis in glioblastoma, liver tumor, and ovarian cancer. No relevant study has been performed on pancreatic cancer to date. The fibrotic area constitutes up to 90% of the tumor volume of pancreatic cancer (Neesse et al., 2015). As the cell type contributing to the vast majority of tumor fibrosis, PSCs play an important role in pancreatic cancer progression (Sousa et al., 2016). Hence, in this report, we focus on its role in PSCs and tumor-stromal interactions. High expression of Arl4c in cancer cells promotes paracrine CTGF signaling, resulting in the induction of autophagy flux in PSCs and subsequent activation of PSCs. Activated PSCs secrete TGFβ1 that activates smad2 signaling in cancer cells and induces cell stemness and drug resistance. YAP is an important mediator downstream of Arl4c, and YAP inhibition restrains the stimulatory effect of Arl4c (Figure 7C).

In summary, our research revealed an important role for Arl4c in pancreatic fibrosis and drug resistance. Strategies targeting Arl4c may be a therapeutic choice for patients with pancreatic cancer, especially for gemcitabine-resistant patients.

### Limitations of the study

In this study, we showed Arl4c is required for cell growth and drug resistance of pancreatic cancer. YAP1 is an important downstream mediator of Arl4c. As we all know, YAP1 regulates cell proliferation in 2D culture system, whereas Arl4c has no effect on cell growth in 2D culture system without PSC co-culture. This may be attributed to other downstream regulator Arl4c, which needs further exploration. Moreover, the PSCs and pancreatic cancer cells co-culture system do not fully model the diverse microenvironments *in vivo*. Also, the results will be much more convincing if the information of gemcitabine-sensitive and gemcitabine-resistant patients' samples were included. Further study will be required to better characterize this function.

## STAR★Methods

### Key resources table

**Table:** 

REAGENT or RESOURCE	SOURCE	IDENTIFIER
**Antibodies**		

Rabbit anti-Arl4c	proteintech	cat# 10202-1-AP; RRID: AB_2058661
Rabbit anti-YAP1	Abcam	cat# ab205270; RRID: AB_2813833
Rabbit anti-p-YAP1	Abcam	cat# ab52771; RRID: AB_2219141
Rabbit anti-SMAD2	proteintech	cat# 12570-1-AP; RRID: AB_2193037
Rabbit anti-p-SMAD2	Abcam	cat# ab280888
Mouse anti-α-SMA	Boster	cat# BM0002; RRID: AB_2811044
Rabbit anti-CTGF	Boster	cat# PB0570
Rabbit anti-Collagen I	Abcam	cat# ab34710; RRID: AB_731684
Mouse anti-CD44	proteintech	cat# 60224-1-Ig; RRID: AB_11042767
Rabbit anti-CD133	proteintech	cat# 18470-1-AP; RRID: AB_2172859
Mouse anti-OCT4	proteintech	cat# 60242-1-Ig; RRID: AB_2881364
Mouse anti-Nanog	proteintech	cat# 67255-1-Ig; RRID: AB_2882529
Rabbit anti-LC3	Abcam	cat# ab192890; RRID: AB_2827794
Mouse anti-PCNA	proteintech	cat# 60097-1-Ig; RRID: AB_2236728
Mouse anti-β-actin	Sigma-Aldrich	cat# A1978; RRID: AB_476692
Goat Anti-mouse dylight 594 IgG antibody	Abbkine	cat# A23410
Goat Anti-Rabbit dylight 488 IgG antibody	Abbkine	cat# A23220; RRID: AB_2737289

**Chemicals, peptides, and recombinant proteins**		

Total RNA Kit II	Qiagen	74204
SB431542	Selleck	S1067
Recombinant Human TGF-β1	peprotech	100-21
Recombinant Human CTGF	peprotech	120-19

### Resource availability

#### Lead contact

Further information and requests for reagents should be directed to and will be fulfilled by the lead contact, Qingyong Ma (qyma56@xjtu.edu.cn).

#### Materials availability

This study did not generate new unique reagents.

### Experimental model and subject details

#### Human tissue specimens and immunohistochemical analysis

Fifty-two pancreatic adenocarcinoma cancer specimens were obtained from the First Affiliated Hospital of Xi’an Jiaotong University between January 2016 and December 2018 under a protocol approved by the relevant ethics committee of the First Affiliated Hospital of Xi’an Jiaotong University. The clinicopathologic data and related Arl4c expressions are summarized in Table S1. All samples were collected according to the informed consent policy. Immunohistochemical staining was performed using a SABC kit (Maxim, Fuzhou, China) according to the standard protocol. The Arl4c staining status was determined according to its cytoplasmic expression in cancer cells. The αSMA staining status was determined according to its cytoplasmic expression in stromal cells. We assigned the percentage score as follows: no cells with cytoplasmic staining (0), 0-25% of cells displayed cytoplasmic staining (1), 25-50% (2), 50-75% (3), and more than 75% of cells displayed cytoplasmic staining (4). We scored the staining intensity as 0 for negative, 1 for weak, 2 for moderate, and 3 for strong. The overall scores were determined by multiplying the percentage score by the intensity score. The overall score of ≤3 was defined as negative, of > 3 and ≤ 6 as weak; of > 6 and ≤ 9 as moderate, and of > 9 as strong.

#### Cell culture and reagents

The human pancreatic cancer cell lines Panc-1 and BxPC-3 were obtained from the Chinese Academy of Science Cell Bank of Type Culture Collection (CBTCCCAS, Shanghai, China). Cells were cultured in proper medium (HyClone, Logan, USA) supplemented with 10% fetal bovine serum (FBS), 100 U/ml penicillin and 100 μg/ml streptomycin at 37°C in a humidified atmosphere containing 5% CO2. Human PSCs were isolated from normal pancreatic tissue from the donor pancreas used for transplantation as previously reported (Bailey et al., 2009). Isolated PSCs were cultured in DMEM/F12 (HyClone, Logan USA) supplemented with 10% fetal bovine serum (FBS), 100 U/ml penicillin and 100 μg/ml streptomycin at 37°C with 5% CO2. For 3D cell culture, a number of pancreatic cancer cells (8000 cells for Panc-1, 16000 cells for BxPC-3) were suspended in 150ul medium. An equal volume of Matrigel was added to the cellular suspension. The cellular/Matrigel suspension was mixed gently and pipetted into each well of a 24-well plate. After solidification, 500ul cancer cell culture medium was added. Cultures were maintained at 37°C and 5% CO2. Cells were harvested at indicated time point using cell recovery solution (Corning, NY) and cells number was calculated.

#### PCs and PSCs coculture model

Direct or indirect coculture systems were used to analyze the interaction between pancreatic cancer cells and PSCs. For the direct coculture system, cancer cells and PSCs mixed at a proportion of 4:1 were seeded in a six-well cell culture plate. For the indirect coculture system, a 0.5 μm pore chamber insert was applied. Tumor cells were seeded in the upper compartment of this chamber, and PSCs were seeded in the lower compartment to explore the effect of cancer cells on PSCs. For the detection of the effect of PSCs on cancer cells, PSCs were seeded in the upper compartment of the chamber, and cancer cells were seeded in the lower compartment. For the intervention with cell conditioned medium, culture medium of cells receiving different treatments was collected and filtered, and then used to treat cells immediately or stored at -80°C.

#### Ethics approval and consent to participate

Ethical approval of the human subjects in our experiment was given by the ethics committee of the First Affiliated Hospital of Medical College, Xi’an Jiaotong University, China. Written informed consents were obtained from the patients participating in the study.

### Methods details

#### ELISA

Panc-1 cells or BxPC-3 cells transfected with or without siArl4c or the Arl4c overexpression plasmid were starved for 48 h, and then the supernatants were collected and centrifuged at 3000 rpm for 10 min to remove particles. PSCs treated with tumor cell conditioned medium for 48 h were starved for 24 h, and supernatants were collected and centrifuged. Secreted levels of soluble CTGF or TGFβ1 were measured using an ELISA Kit (R&D Systems, USA) according to the manufacturer’s protocol. A standing curve was used to calculate TGFβ1 or CTGF concentrations based on the OD value of the samples at 450 nm.

#### Oil red O staining

PSCs were washed with PBS 3 times and then fixed with 10% formalin. After rinses with absolute propylene glycol, cells were stained with a prewarmed Oil red O solution for 20 min in 60°C ovens. After differentiation with 75% ethanol, the cells were subjected to nuclear staining with Mayer’s hematoxylin. The cellular staining level was imaged using a microscope (Nikon Instruments Inc.).

#### Immunofluorescence staining

Cells that received different treatments were washed with PBS 3 times and then fixed with 10% formalin at 37°C for 30 min. Cells were then permeabilized with 0.3% Triton-100 in PBS for 10 min at 37°C followed by 2% bovine serum albumin (BSA) blockade for 1 h. After an incubation with the primary antibodies overnight at 4°C, the cells were then incubated with the corresponding secondary antibodies. Nuclear staining was performed using DPAI at a working concentration of 100 ng/ml. The results were imaged using a ZEISS Instruments confocal microscope.

#### Cell transfection

For gene Arl4c knockdown, two siRNAs were designed by GenePharm (Shanghai, China): siArl4c#1: GUAGGUCAUUAUCACACAATT and siArl4c#2: GGCAACAUCUCCUCUAA-CATT. Sense sequences of the siRNA oligos and negative control were as follows: siAtg5: GACCUUUCAUUCAGAAGCUTT, siAtg7: CAGCCUGGCAUU-UGAUAAATT, and negative control siRNA, UUCUCCGAACGUGUCACGUTT. The Arl4c overexpression plasmid was purchased from GeneChem Co., Ltd. (Shanghai, China). For siRNA or plasmid transfection, 1.5×10ˆ5 cells were seeded in 6-well plates and cultured overnight. Cells were starved for 8 h before transfection. Then, cells were transfected with the siRNA at a working concentration of 100 nM or with 2 μg of DNA mixed gently with 4 μl of Lipofectamine 2000. Cells were cultured in fresh medium supplemented with 10% FBS 8 h later. The cell transfection efficiency was confirmed using qPCR and western blotting. For autophagy detection, ad-mRFP-GFP-LC3 was purchased from HanBio Technology Co., Ltd., China. Cells were transfected according to the manufacturer’s recommendation.

#### Western blotting

Total cellular proteins were extracted with RIPA lysis buffer (Healforce, Xi’an, China), and the protein concentration was determined using a BCA protein assay kit (Bio-Rad, Hercules, CA, USA) according to the manufacturer’s protocol. Then, standard western blotting was performed. Protein expression was visualized using an ECL substrate kit and imaged with a ChemiDoc XRS imaging system (Bio-Rad, USA). β-Actin was used as a loading control.

#### qPCR

Total RNA was extracted using a Fastgen200 RNA isolation system (Fastgen, Shanghai, China) according to the manufacturer’s protocol. Then, 1 μg of RNA was reverse-transcribed into cDNAs using a Prime Script RT reagent kit (TaKaRa, Dalian, China). Expression levels of the Arl4c, YAP, CTGF, Shh, VEGF, PDGF, and TGFβ1 mRNAs were quantified using real-time PCR with a SYBR Green Kit (TaKaRa), as previously reported. Β-ACTIN was selected as a normalization control. The primer sequences for Arl4c, YAP, CTGF, Shh, VEGF, PDGF, TGFβ1 and β-actin are listed in Table S2.

#### Cell counting Kit-8

Cell viability was measured using Cell Counting Kit-8 (CCK-8) according to the manufacturer’s protocol. Briefly, 1500 PSCs were seeded in 96-well plates and incubated for the indicated times. Before testing, 10 μl of CCK-8 solution were added to each well of the plate. The plate was incubated for 4 h at 37°C, and the optical density (OD) was measured at 450 nm using a multifunctional microplate reader (POLARstar OPTIMA; BMG, Offenburg, Germany).

#### Tumor sphere formation assays

Tumor cells were harvested and washed 2 times with PBS. Then, the cells were resuspended in a small volume, and the cells were counted using trypan blue. A 20 μl suspension containing 1000 cells and 4 ml of tumor sphere medium (20 ng/ml EGF, 20 ng/ml FGF, and 1×B27 in DMEM/F12 medium) were mixed gently and then added to ultralow attachment 6-well plates. Cells were cultured in an incubator set to 37°C supplied with 5% CO2. One week later, tumor sphere formation was assessed and imaged using a microscope (Nikon Instruments Inc.).

#### Cell invasion assay

Cells invasive abilities were tested using Matrigel Transwell assay. Chambers were pre-coated with Matrigel (BD Biosciences, USA). Conditioned medium of cancer cells with different Arl4c expression levels were collected. Approximately 5×104 PSC cells suspended in 200 μl of serum-starved medium were seeded into the upper chamber. Then, 500 μl of cancer cells conditioned medium was added to the lower chamber. After 24 h, the cells were washed with PBS 3 times, fixed with 4% paraformaldehyde, and stained with crystal violet. Dissolved crystal violet with 33% acetic acid under 570 nm from three independent experiments and cells invasive abilities were quantified.

### Quantification and statistical analysis

Statistical analyses were performed using Graphpad Prism 7. All experiments were repeated three times independently. Comparisons between groups were analyzed using appropriate statistical methods including Student’s t test and one-way ANOVA test. The Spearman correlation test was used to assess the relationships of gene expression. The data are shown as the mean with error bars showing ±SEM. A P value of less than 0.05 was considered statistically significant.

## Data Availability

All data produced in this study are included in the published article and its supplemental information, or are available from the lead contact upon request. This paper does not report original code. Any additional information required to reanalyze the data reported in this work paper is available from the lead contact upon request.
