# Supplementary material for: Arl4c promotes the growth and drug resistance of pancreatic cancer by regulating tumor-stromal interactions
Source: iScience. 2021 Nov 3;24(12):103400. doi: 10.1016/j.isci.2021.103400 (PMC8609020; doi:10.1016/j.isci.2021.103400)
Supplement: Document S1. Tables S1 and S2 [file mmc1.pdf]

iScience, Volume *24*

Supplemental information

Arl4c promotes the growth

and drug resistance of pancreatic cancer

by regulating tumor-stromal interactions

Xin Chen, Yanzhen Zhang, Weikun Qian, Liang Han, Wei Li, Wanxing Duan, Zheng Wu, Zheng Wang, and Qingyong Ma

Table S1: The relationship between expression of Arl4c and clinical pathological features in pancreatic cancer, Related to Figure 7.

	Samples	Arl4c expression n (%)				P value◇
	(n)	None	Weak	Moderate	Strong	
sex						0.9019
Male	33	3（5.77）	8（15.38）	14（26.92）	8（15.38）	
Female	19	3（5.77）	4（7.69）	8（15.38）	4（7.69）	
Age						0.56456
<55	28	4（7.69）	8（15.38）	11（21.15）	5（9.6）	
>55	24	2（3.85）	4（7.69）	11（21.15）	7（13.46）	
Histological grade						0.26321
Well	9	3（5.77）	2（3.85））	3（5.77）	1（1.92）	
Moderate	33	2（3.85））	8（15.38）	13（25%）	10（19.23）	
Poor	10	1（1.92）	2（3.85）	6（11.54）	1（1.92）	
TNM stage (AJCC)						0.01615*
I	2	1（1.92）	1（1.92）	0（0）	0（0）	
II	39	5（9.6）	11（21.15）	18（34.62）	5（9.6）	
III	6	0（0）	0（0）	3（5.77）	3（5.77）	
IV	5	0（0）	0（0）	1（1.92）	4（7.69）	
pT status						0.21467
T1	3	2（3.85）	1（1.92）	0（0）	0（0）	
T2	35	3（5.77）	7	16（30.77）	9（17.31）	
T3	7	0（0）	2（3.85）	3（5.77）	2（3.85）	
T4	7	1（1.92）	2（3.85）	3（5.77）	1（1.92）	
pN status						0.67789
NO	42	4（7.69）	9（17.31）	19（36.54）	10（19.23）	
N1	10	2（3.85）	3（5.77）	3（5.77）	2（3.85）	
pM status						0.00995*
M0	45	6（11.54）	12（23.08）	20（38.46）	7（13.46）	
M1	7	0（0）	0（0）	2（3.85）	5（9.6）	

◇χ^2 test. *，*P* <0.05.

Table S2: Primers sequences for real-time PCR analysis, Related to STAR Methods.

**Genes**	**Primer Sequences**
YAP	Forward:5’-TCCCAGATGAACGTCACAGC-3’
	Reverse: 5’-TCATGGCAAAACGAGGGTCA-3’
shh	Forward:5’- GCGACTTCCTCACTTTCCTG -3’
	Reverse: 5’- CCGGTTGATGAGAATGGTG -3’
VEGF	Forward:5’- AAGCCAGCACATAGGAGAGATGA-3’
	Reverse: 5’- TCTTTCTTTGGTCTGCATTCACA-3’
PDGF	Forward:5’- CCCCTGCCCATTCGGAGGAAGAG-3’
	Reverse: 5’- TTGGCCACCTTGACGCTGCGGTG -3’
TGFβ1	Forward:5’- CCCAGCATCTGCAAAGCTC-3’
	Reverse: 5’- GTCAATGTACAGCTGCCGCA-3’
CTGF	Forward:5’-CTTTGGCCCAGACCCAACTA-3’
	Reverse: 5’-GGCTCTGCTTCTCTAGCCTG-3’
β-ACTIN	Forward:5’- AGCGAGTATCCCCCAAAGTT -3’
	Reverse: 5’- GGGCACGAAGGCTCATCATT -3’
